# Influence of Sleep Quality on Recovery and Performance in Endurance and Ultra-Endurance Runners: Sex Differences Identified Through Hierarchical Clustering

**DOI:** 10.3390/healthcare13070812

**Published:** 2025-04-03

**Authors:** Julia Pagotto Matos, Larissa Quintão Guilherme, Samuel Gonçalves Almeida da Encarnação, Luciano Bernardes Leite, Pedro Forte, Ana Claudia Pelissari Kravchychyn, Paulo Roberto dos Santos Amorim, Helton de Sá Souza

**Affiliations:** 1 Department of Physical Education, Universidade Federal de Viçosa, Viçosa 36570-900, Brazil; julia.pagotto@ufv.br (J.P.M.); larissa.guilherme@ufv.br (L.Q.G.); luciano.leite@ufv.br (L.B.L.); pramorim@ufv.br (P.R.d.S.A.); 2 Department of Sports, Instituto Politécnico de Bragança, 4560-708 Bragança, Portugal; samuel01.encarnacao@gmail.com; 3 Department of Physical Education, Sport and Human Movement, Universidad Autónoma de Madrid (UAM), Ciudad Universitaria de Cantoblanco, 28049 Madrid, Spain; 4 CI-ISCE, Instituto Superior de Ciências Educativas do Douro (ISCE Douro), 4560-000 Penafiel, Portugal; 5 Department of Sports, Instituto Superior de Ciências Educativas do Douro (ISCE Douro), 4560-000 Penafiel, Portugal; 6 Research Center for Active Living and Wellbeing (LiveWell), Instituto Politécnico de Bragança, 5300-253 Bragança, Portugal; 7 Department of Nutrition, Universidade Federal de Viçosa, Viçosa 36570-900, Brazil; ana.pelissari@ufv.br; 8 Department of Biomedical and Biotechnological Sciences, School of Medicine, University of Catania, 95123 Catania, Italy; 9 Research Center on Motor Activities (CRAM), University of Catania, 95123 Catania, Italy

**Keywords:** sleep quality, endurance athletes, ultra-endurance, recovery, athletic performance

## Abstract

**Background:** Assessing sleep quality is essential in sports science, particularly in ultra-endurance sports, where recovery is critical for performance and health. **Objective:** This study aimed to identify sleep quality patterns among endurance and ultra-endurance athletes using hierarchical clustering analysis, with comparisons by sex and modality. **Method:** Data were collected during the La Misión Brasil competitions in 2023 and 2024, using the Pittsburgh Sleep Quality Index (PSQI). The questionnaire was emailed to all registered runners two weeks before the event. A total of 490 athletes participated, including 276 men (mean ± SD age: 43 ± 11 years) and 214 women (mean ± SD age: 43 ± 13 years). Statistical analyses included Cohen’s d and r effect sizes, and a 95% confidence interval for hypothesis testing. Residuals between-cluster proportions were assessed within a range of −3.3, ensuring a 99.7% confidence level for significant differences. **Results:** The results showed that endurance runners had better sleep quality, with most scoring low on the PSQI. In contrast, ultra-endurance athletes displayed greater variability, with a higher prevalence of poor sleep quality, particularly in women. **Conclusions:** The hierarchical clustering method effectively identified distinct sleep patterns, providing insights into the dynamics of recovery and performance. These findings highlight the impact of increased physical and psychological demands in ultra-endurance sports and emphasize the need for tailored sleep monitoring strategies to optimize the recovery and performance of athletes.

## 1. Introduction

Trail running has experienced significant growth in popularity in recent years, establishing itself as a prominent endurance sport modality on a global scale [1,2,3]. Recreational practice and participation in organized events have expanded at an average annual rate of 15% over the past decade [4]. Between 2013 and 2019, the International Trail Running Association recorded more than 25,700 races in 195 countries, highlighting the global reach of this sport [5]. In this context, ultramarathons, defined as races exceeding the distance of a marathon (>42.195 km) and often held in remote and natural areas, have seen a 1676% increase in global participation between 1996 and 2018 [6,7].

Ultramarathons demand exceptional physical and mental effort, influenced by the complex interaction of physiological, psychological, and environmental parameters [8,9]. La Misión Brazil, a trail running competition held in the Serra da Mantiqueira, Minas Gerais, is considered one of the most challenging races in Brazil due to its demanding course, which includes steep elevation gains, uneven terrain, elevation profiles, and climatic variations that play a crucial role in participant performance [3,10,11]. These obstacles not only require detailed training planning but also emphasize the importance of proper recovery, with sleep being a central element in the physical and mental restoration of athletes.

In recent years, sleep quality has emerged as a key variable in sports science, given its crucial role in fatigue recovery, physiological and psychological restoration, and overall athletic performance, particularly in endurance sports [12,13,14]. However, it is important to distinguish between sleep duration and sleep quality. While duration refers to the total time spent sleeping, sleep quality encompasses multiple aspects, such as efficiency, sleep latency, wakefulness after sleep onset, and disturbances, all of which influence the body’s ability to recover and adapt to training loads [15,16,17]. In ultra-endurance contexts, factors such as accumulated fatigue, anxiety, and environmental conditions can negatively impact sleep quality, further complicating recovery and performance optimization [18,19,20].

Additionally, individual characteristics, such as sex, may influence sleep patterns. Studies suggest that women experience greater variability in sleep quality, potentially due to hormonal fluctuations across the menstrual cycle, while men tend to have more stable sleep patterns, which may favor recovery [21,22]. Moreover, sleep architecture, including the distribution of sleep stages, may differ between endurance and ultra-endurance athletes, affecting their ability to recover efficiently [23,24]. Given these potential differences, further research is needed to better understand how sex and training demands interact to influence sleep quality and recovery, particularly in ultra-endurance sports, where sleep can be a determining factor for both performance and long-term health outcomes.

Despite the growing body of evidence on the importance of sleep for athletic performance, the use of advanced techniques may contribute to a better understanding of sleep patterns in ultramarathon runners. Hierarchical clustering is a valuable tool for identifying subgroups with similar characteristics and key factors influencing sleep quality [25]. Unlike traditional methods, it enables data-driven classification of athletes, revealing hidden patterns within complex datasets [26]. While widely applied in fields like public health, its use in sports science remains limited, despite its potential to analyze multifactorial variables such as sleep and recovery. Recent studies have highlighted its effectiveness in categorizing athletes based on their physiological and training profiles, reinforcing its relevance in sports research [26,27,28].

However, despite the advancements in sleep analysis, key gaps remain in the literature. There is still a lack of studies comparing sleep quality between endurance and ultra-endurance athletes, as well as a limited understanding of how sex differences influence sleep in these populations. Given the unique demands of ultra-endurance sports, further research is needed to explore how training load, physiological adaptations, and psychological stress interact to shape sleep patterns in different athletic groups. A clearer understanding of these factors could provide valuable insights into sports science, aiding the development of targeted strategies to enhance athlete recovery and performance.

We hypothesize that sleep quality patterns will differ between endurance athletes and ultra-endurance athletes, as the higher training load required for ultra-endurance events may contribute to greater variability and poorer sleep quality in these athletes. Additionally, we expect sex-based differences, with female athletes experiencing more sleep disturbances, potentially influenced not only by hormonal fluctuations and psychological factors but also by greater social demands. Addressing these gaps, the present study aims to investigate sleep quality patterns in trail and mountain runners, analyzing male and female athletes of different ages and experience levels participating in a specific endurance event. Using the hierarchical clustering method, this study seeks to identify specific sleep quality patterns among male and female endurance and ultra-endurance athletes.

## 2. Materials and Methods

### 2.1. Study Design

This is an observational and cross-sectional study conducted with endurance and ultra-endurance athletes during the La Misión Brasil trail and mountain running competition, held in August 2023 and 2024. The study was approved by the Research Ethics Committee of the Federal University of Viçosa (CAAE: 48570921.4.0000.5153, approval number: 4.911.679) and was conducted in accordance with the Helsinki Declaration, following all ethical guidelines and with the informed consent of all participants.

### 2.2. Participants

The study population consisted of approximately 5464 endurance and ultra-endurance trail and mountain runners who participated in the La Misión Brazil race, competing in distances of 7 km, 15 km, 25 km, 35 km, 55 km, and 80 km. The study sample included 490 athletes, of whom 276 were men (mean ± SD, age: 43 ± 11 years, body mass: 73 ± 10.2 kg, height: 1.7 ± 0.1 m, body mass index: 24 ± 3 kg·m^2^) and 214 women (mean ± SD, age: 43 ± 13 years, body mass: 60 ± 10 kg, height: 1.6 ± 0.1 m, body mass index: 23 ± 4 kg·m^2^), as shown in Table 1. The statistical power for the categorical analysis was calculated a priori using the R programming language [29]. For this purpose, the parameters of the number of groups predefined by the hierarchical clustering algorithm, the alpha of *p* < 0.05, and a moderate effect size adjusted according to the degrees of freedom were set to calculate statistical power. In the same way, for continuous variables, the statistical power to independent samples statistics was calculated, setting the parameters of a moderate effect size (d = 0.5) according to Cohen’s guidelines, in a 95% confidence interval of a two-sided hypothesis [30]. Thus, only tests with statistical power values ≥ 0.8 were considered reportable outputs [30].

### 2.3. Procedures

Data were collected during the 2023 and 2024 editions of La Misión Brasil, held from 17–20 August 2023, and 15–18 August 2024, in the city of Passa Quatro, Minas Gerais, Brazil. With the consent and support of the competition organizers, a list of registered participants, including the athletes’ personal emails, was provided, allowing the researchers to contact them. In the two weeks preceding each competition, a self-administered questionnaire was emailed to all registered athletes to collect information on anthropometrics and body composition and to subjectively assess participants’ sleep quality using the Pittsburgh Sleep Quality Index (PSQI) [28].

All athletes received detailed instructions regarding the procedures for measuring body mass and height. However, since the measurements were performed autonomously by the volunteers, this method may present limitations regarding data accuracy.

### 2.4. Anthropometrics and Body Composition

Body weight and body height were obtained using a self-reported questionnaire designed to characterize the sample and gather anthropometric data. These measures were then used to calculate the BMI (Body Mass Index) using the standard formula: weight (kg) divided by height squared (m^2^).

### 2.5. Pittsburgh Sleep Quality Index (PSQI)

Sleep quality was assessed using the PSQI, a questionnaire comprising 19 items that evaluate subjective sleep quality [31]. The PSQI is a self-administered tool used to subjectively assess sleep quality and potential sleep disorders in the past month. Due to its reliability and ease of understanding, this instrument is widely employed in epidemiological studies and clinical research [31,32]. The PSQI has been translated and validated in Brazilian Portuguese [32]. The PSQI consists of 19 items organized into 10 questions, which comprise seven components that contribute to the global index score. These components assess different aspects of sleep: subjective sleep quality, sleep latency, sleep duration, habitual sleep efficiency, presence of sleep disturbances, use of sleep medication, and daytime dysfunction. The global PSQI score is obtained by summing the scores of these components and is classified as follows: 0 to 4 indicates good sleep quality, 5 to 10 suggests poor sleep quality, and scores above 11 may indicate the presence of sleep disorders. For this study, only the global PSQI score was used, which ranged from 0 to 21 points, with higher scores indicating poorer sleep quality.

Data were collected and initially tabulated using Excel (version 2024). Subsequently, statistical analysis was performed using the R programming language with specific packages for data processing and analysis.

### 2.6. Hierarchical Clustering Model

To identify groups of runners according to their sleep quality scores in the PSQI, a hierarchical clustering method was applied [33]. For this purpose, all analyses were performed in R, a statistical computing programming language, where the library “dplyr” was activated to preprocess the data of PSQI scores in a data frame file, where it was possible to perform the clustering [34]. Additionally, the dataset used was normalized in the range of −1,1 to avoid overfitting during the clustering calculation [35]. Next, activating the library “cluster” the distance between points was calculated using the Euclidean distance, and hierarchical clustering was performed using the method “complete”, which refers to the complete linkage, which works by merging small clusters into principal clusters and dividing large clusters into individual ones [33]. The best number of clusters was calculated using the silhouette score (SS), the within-cluster dispersion was calculated with the within-cluster sum of squares (WCSS), the distances of the clusters were calculated with the between-cluster sum of squares (BCSS), and the proportion of division of between clusters under the within-cluster distributions was calculated with the BCSS/WCSS ratio. The sum of BCSS and WCSS defined the total sum of squares (TSS), and the total explanation of the cluster distributions by the hierarchical clustering method was calculated using the BCSS/TSS ratio [33]. Dendrograms were plotted by activating the library “ggdendro” [36].

### 2.7. Statistical Analysis

Initially, the Shapiro-Wilk test was performed to check the data distributions for the statistics regarding the participants’ characteristics. Considering a 95% confidence interval, the independent *t*-test or Wilcoxon signed-rank test was employed for parametric and non-parametric data, respectively [37]. For statistically significant differences, Cohen’s d and r effect sizes were calculated considering Cohen’s cut-offs of small = 0.20, moderate = 0.5, and large = 0.8 were set to measure the meaningfulness of the differences [30]. The point-biserial correlation was applied to identify relationships between sex and PSQI scores, respecting Cohen’s effect size guidelines of 0.10 = small, 0.30 = moderate, and 0.50 = large [30]. The chi-square goodness of fit was calculated to measure the differences in the proportions of runners within the previously established clusters by the hierarchical clustering model. Thus, a 95% confidence interval was considered to accept the alternative hypothesis regarding significant differences between clusters. If significant differences were found, the residuals between the proportions of the n-analyzed clusters were considered in a range of −3,3, where an interval confidence of 99.7% was reached to ensure statistically significant differences to accept the alternative hypothesis [37]. Cohen’s Omega (ω) was calculated to measure the effect size of the correlations, attending the cut-offs of 0.10 = small, 0.30 = moderate, and 0.50 = large, according to Cohen [30]. All statistical procedures were performed using the R programming language [29].

## 3. Results

Table 2 shows the correlation analysis results of the overall classes of runners, where the female sex was positively correlated with PSQI scores and the male sex was inversely correlated with PSQI scores, indicating that women were more subjected to poor sleep quality. Nevertheless, the effect sizes were small, highlighting the large influence of sex on athletes’ sleep quality.

Table 3 and Table 4 show the correlation results for endurance and ultra endurance athletes by sex, respectively. In this group, despite the correlations, the coefficients were slightly larger than those of the overall population of athletes, and the effect sizes were maintained within the small cut-off.

### 3.1. Female Endurance Athletes

**Figure 1:**
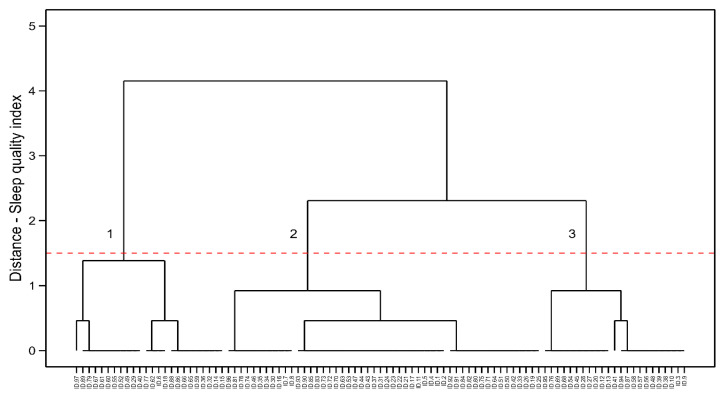
HC visualization in female endurance running athletes. Cluster 1: the poorer sleep quality indexes, Cluster 2: the mid-levels of sleep quality and Cluster 3: the best sleep quality indexes.

Figure 1 shows the results for the group of female endurance athletes. The hierarchical clustering model identified three clusters [SS = 0.62, WCSS = 13.17, BCSS = 5695.448, TSS = 5708.626, BCSS/WCSS = 432.188, BCSS/TSS = 0.99%], with Cluster 1 being the one with athletes scoring 7–10 points in the PSQI (n = 24, 25%), Cluster 2 presented athletes scoring 4–6 points (n = 50, 52%), and Cluster 3 scoring 1–3 points (n = 23, 24%). The X2 test verified significant differences between the clusters [X2 = 14.5, df = 2, *p* = 0.0007], and the post hoc test revealed that Cluster 2 had a significantly higher number of subjects than Clusters 1 and 3 [residuals = 3.114].

### 3.2. Female Ultra-Endurance Athletes

**Figure 2:**
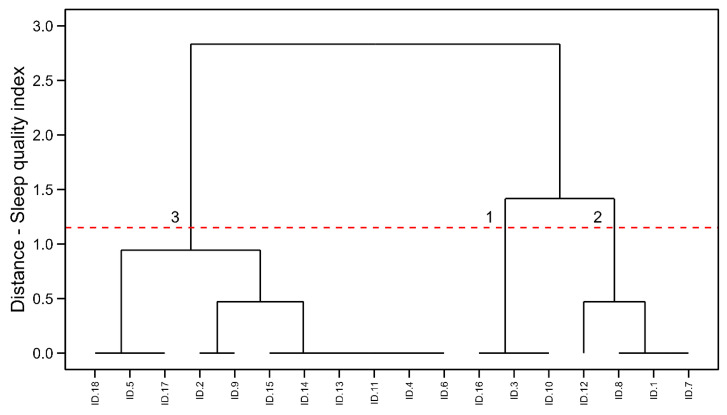
HC visualization in female ultra-endurance running athletes. Cluster 1: the poorer sleep quality indexes, Cluster 2: the mid-levels of sleep quality and Cluster 3: the best sleep quality indexes.

Figure 2 shows the hierarchical clustering results for female ultra-endurance running athletes. Similar to the first group of female athletes, the hierarchical clustering model identified three clusters [SS = 0.76, WCSS = 5.659, TSS = 958.33, BCSS = 952.674, BCSS/WCSS = 168.344, BCSS/TSS = 0.99%]: Cluster 1 with athletes scoring 8 points in PSQI (n = 3, 17%), Cluster 2 with individuals scoring 5–6 points (n = 4, 22%), and Cluster 3 scoring 2–4 points (n = 11, 61%). Owing to reduced statistical power [0.24], between-group comparisons using the X2 statistics were not possible.

### 3.3. Male Endurance Athletes

**Figure 3:**
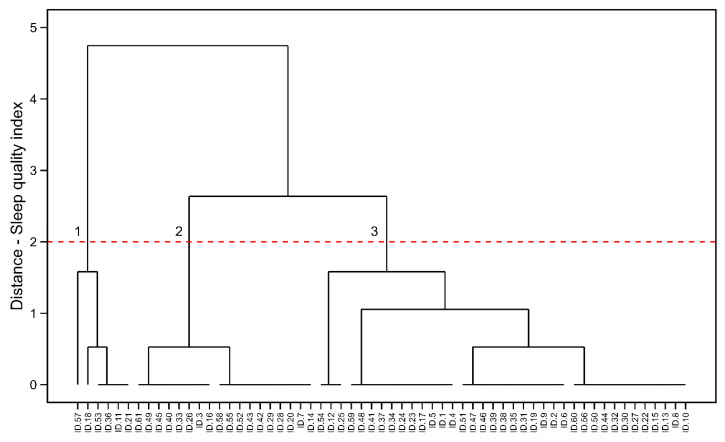
HC visualization in male endurance running athletes. Cluster 1: the poorer sleep quality indexes, Cluster 2: the mid-levels of sleep quality and Cluster 3: the best sleep quality indexes.

Figure 3 shows the hierarchical clustering results for male endurance running athletes. The model also identified three clusters within the dataset [SS = 0.58, WCSS = 12.641, BCSS = 2235.772, TSS = 2248.413, BCSS/WCSS = 176.862, and BCSS/TSS = 0.99%]. Cluster 1, the athletes scored 7–10 points in the PSQI (n = 6, 10%), the athletes within Cluster 2 scored 5–7 points (n = 18, 30%), and the athletes from the Cluster 3 scored 1–4 points (n = 37, 61%). Calculating the X2 statistics between clusters was also impossible due to the reduced statistical power [0.69].

### 3.4. Male Ultra-Endurance Athletes

**Figure 4:**
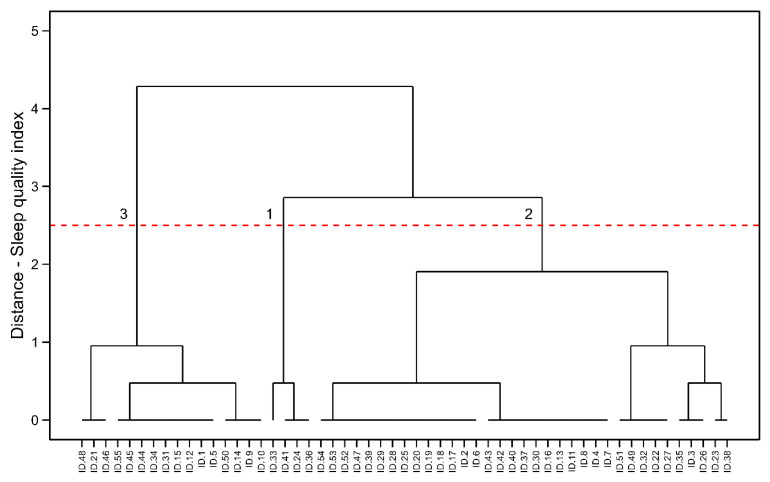
HC visualization in male ultra-endurance running athletes. Cluster 1: the poorer sleep quality indexes, Cluster 2: the mid-levels of sleep quality and Cluster 3: the best sleep quality indexes.

Figure 4 shows the results for male ultra-endurance athletes. Hierarchical clustering also identified three clusters [SS = 0.52, BCSS = 1773.203, WCSS = 13.629, TSS = 1786.833, BCSS/WCSS = 130.0967, BCSS/TSS = 0.99%]. Cluster 1 was composed of participants who scored 9–10 points in the PSQI (n = 3, 4%), the Cluster 2 presented participants with scores between 1–3 points (n = 36, 66%), and the Cluster 3 presented participants with scores ranging 4–8 points (n = 16, 30%). It was also impossible to calculate between-cluster statistics because the sample of 55 participants produced a statistical power of 0.64.

## 4. Discussion

This study aimed to identify sleep quality patterns in endurance and ultra-endurance athletes using the hierarchical clustering method, with analyses stratified by sex and modality. The results revealed significant differences in sleep patterns, reflecting the specific characteristics of each group and their unique athletic demands. These findings reinforce the growing body of evidence that links training load, psychological stress, and environmental factors to sleep disturbances in high-performance sports.

Among endurance runners, a higher proportion of participants in clusters with low PSQI scores indicated satisfactory sleep quality. This pattern is consistent with studies suggesting that endurance athletes often manage to balance their training routines with adequate periods of rest and recovery, contributing to regular sleep patterns [38,39,40]. Furthermore, training in endurance modalities generally involves moderate training loads, allowing for more efficient recovery and reducing the accumulated physiological impact [41,42]. Previous research has shown that moderate training intensity may positively influence sleep architecture by promoting deeper sleep stages, which are essential for muscle repair and cognitive function [43,44,45]. These factors may explain the predominance of clusters with better sleep scores in this group. Nevertheless, trainers must consider the clusters with athletes at higher risk of sleep disorders during the athletes’ periodization to avoid the increased likelihood of sleep disorders and reductions in running performance.

In contrast, the results for ultramarathon runners indicated greater variability in PSQI scores, with a proportion of athletes grouped into clusters of reduced sleep quality. This trend is widely documented in the literature, which highlights the negative impact of long-duration events and high-intensity training in ultra-endurance modalities [46,47,48,49]. Chronic physiological stress resulting from intense training can compromise recovery and lead to fragmented and less efficient sleep patterns [11,50,51]. These findings reinforce the complexity of the demands placed on ultramarathon runners and highlight the need for specific approaches to minimize the negative impact of ultramarathon training on sleep quality, which is essential for performance and long-term health.

Beyond training load and psychological stress, factors such as training schedules, training intensity, nutrition, caffeine consumption, and travel may interfere with sleep quality in endurance and ultra-endurance athletes, potentially compromising their recovery and performance [52,53,54]. Therefore, incorporating structured sleep hygiene protocols into training and recovery may help mitigate these effects. Maintaining a consistent sleep schedule, avoiding stimulants, minimizing evening screen exposure, and using relaxation techniques may improve sleep quality among athletes [55].

Among female ultra-endurance runners, higher PSQI scores than those of men indicate overall lower sleep quality, consistent with studies highlighting sex differences in sleep patterns. Hormonal fluctuations throughout the menstrual cycle, particularly during the luteal phase, are known to negatively impact women’s sleep quality, leading to lower efficiency and increased wakefulness [56,57,58]. Additionally, psychosocial factors, such as the higher prevalence of anxiety and stress often reported in female athletes, can exacerbate sleep-related difficulties [21,59]. Such evidence suggests that interventions specifically tailored to the physiological and psychological needs of female athletes could be beneficial to this group during the training process.

Among male runners, both in endurance and ultra-endurance modalities, sleep patterns exhibited greater homogeneity, with a higher concentration of individuals in clusters indicating better sleep quality than in female runners. Similar findings have been reported in previous studies, which also observed better sleep quality among male athletes than among their female counterparts [20,21]. This trend may be related to hormonal differences and physiological characteristics that positively influence sleep in men, such as greater stability of circadian rhythms and lower susceptibility to sleep disorders [60,61]. However, even within these groups, clusters with intermediate scores were observed, particularly among ultramarathon runners, which may be attributed to factors such as higher training loads, accumulated sleep deprivation, and irregular schedules, which are common among elite athletes during training and competition periods [62,63]. These findings highlight the importance of considering not only sex-related differences but also the cumulative effects of prolonged endurance training on sleep patterns, which may require individualized recovery strategies for male and female athletes alike. Thus, the hierarchical clustering classification indicates that there is a group of male athletes with an increased likelihood of moving to clusters with a higher risk of sleep disorders.

The hierarchical clustering method effectively identified distinct sleep quality patterns in endurance and ultra-endurance athletes. The high BCSS/TSS index (99%) confirmed the robustness of the model, demonstrating its suitability for capturing variations in the analyzed data. These findings reinforce the potential of hierarchical clustering as an analytical tool in sports science, particularly for identifying factors associated with sleep disturbance in athletes.

This highlights the need for individualized strategies to monitor and optimize sleep, especially in ultra-endurance athletes, in whom sleep-related challenges are more pronounced. Implementing targeted interventions, such as sleep hygiene practices, which have gained prominence in the sports context, is considered an economical, non-invasive, and effective method capable of improving subjective and objective sleep parameters in athletes [64,65,66,67]. Other adjustments, such as better control over training load [68] and psychological support, may be essential for mitigating the impact of accumulated stress on recovery and performance [69].

However, this study had some limitations. Its cross-sectional design prevents the assessment of sleep variability over time, and reliance on subjective measures (PSQI) without objective assessments, such as actigraphy or polysomnography, limits the precision of sleep evaluation. Additionally, the small sample size of certain subgroups restricted the statistical analysis and generalizability. The lack of comparison between endurance and non-endurance athletes further limits the insights into the specific impact of prolonged physical exertion on sleep quality. Moreover, recovery-related physiological and biochemical markers were not assessed, which constrained the exploration of their relationship with sleep patterns.

Future research should address these limitations by incorporating longitudinal designs, larger sample sizes, and objective sleep measurements. Comparing endurance and non-endurance athletes while integrating variables related to training load and physiological recovery could provide a more comprehensive understanding of the factors influencing the sleep quality in this population.

## 5. Conclusions

This study identified distinct sleep quality patterns in endurance and ultra-endurance runners. Endurance runners exhibited better sleep quality, whereas ultramarathon runners showed greater variability, with a higher prevalence of poor sleep quality, particularly in women. These findings underscore the importance of individualized strategies for monitoring and improving sleep, taking into account the specific characteristics of each group and sports modality.

## Figures and Tables

**Table 1:** Represents the participants’ characteristics.

	Male (n = 276)	Female (n = 214)	W	*p*	d
Age	43 (11)	43 (13)	29,296	0.87	0.006
Body weight	73 (10.2)	60 (10)	7709	2.2 × 10^−16^	0.66
Body height	1.7 (0.1)	1.6 (0.1)	7964.5	2.2 × 10^−16^	0.63
BMI	24 (3)	23 (4)	20,689	1.016 × 10^−8^	0.26

Note. Data are presented as median and interquartile range. W: statistics for the Wilcoxon signed-rank test; *p*: *p*-value in a 95% confidence interval; d: Cohen’s d for effect size calculation.

**Table 2:** Correlation results between sex and PSQI in all running categories.

Sex	t	df	*p*	CI	Correlation
Male (n = 276)	2.463	488	0.01	0.023–0.124	−0.11
Female (n = 214)	2.463	488	0.01	0.023–0.124	0.11

Note. Data are presented as absolute values. t: t-statistics for the point-biserial correlation; *p*: *p*-value in a 95% confidence interval; CI: 95% confidence interval.

**Table 3:** Correlation results between sex and PSQI scores in endurance athletes.

Sex	t	df	*p*	CI	Correlation
Male (n = 115)	2.857	156	0.005	−0.366, −0.06	−0.22
Female (n = 32)	2.463	156	0.005	0.023, 0.124	0.22

Note. Data is presented in absolute values. t: t-statistics for the point-biserial correlation, *p*: *p*-value in a 95% confidence interval, CI: 95% confidence interval.

**Table 4:** Outputs the correlation results of ultra-endurance athletes by sex. In this group, despite the correlations, the coefficients were slightly larger than those of the overall population of athletes, and the effect sizes were kept within the small cut-off.

Sex	t	df	*p*	CI	Correlation
Male (n = 161)	0.496	71	0.62	−0.173, 0.285	0.06
Female (n = 182)	0.496	71	0.62	−0.173, 0.285	0.06 ^#^

Note. Data are presented as absolute values. t: t-statistics for the point-biserial correlation; *p*: *p*-value in a 95% confidence interval; CI: 95% confidence interval. #: Result with low statistical power (power = 0.39).

## Data Availability

The data supporting the findings of this study are available from the corresponding author (P.F.) upon reasonable request.

## Abbreviations

The following abbreviations are used in this manuscript:

BCSS	Between-Cluster Sum of Squares
BMI	Body Mass Index
CAAE	Certificate of Presentation for Ethical Assessment
ITRA	International Trail Running Association
Km	Kilometer(s)
PSQI	Pittsburgh Sleep Quality Index
SD	Standard deviation
SS	Silhouette Score
TSS	Total Sum of Squares
UFV	Universidade Federal de Viçosa
WCSS	Within-Cluster Sum of Squares
X^2^	Chi-square test
